# Nanoparticle-Assisted Diagnosis and Treatment for Abdominal Aortic Aneurysm

**DOI:** 10.3389/fmed.2021.665846

**Published:** 2021-07-07

**Authors:** Li Yin, Kaijie Zhang, Yuting Sun, Zhenjie Liu

**Affiliations:** ^1^Department of Vascular Surgery, The Second Affiliated Hospital of Zhejiang University School of Medicine, Hangzhou, China; ^2^Department of Cardiology, Sir Run Run Shaw Hospital of Zhejiang University School of Medicine, Hangzhou, China; ^3^Department of Orthopaedic Surgery, Sir Run Run Shaw Hospital of Zhejiang University School of Medicine, Hangzhou, China

**Keywords:** nanoparticles, abdominal aortic aneurysm, imaging, treatment, diagnosis

## Abstract

An abdominal aortic aneurysm (AAA) is a localized dilatation of the aorta related to the regional weakening of the wall structure, resulting in substantial morbidity and mortality with the aortic ruptures as complications. Ruptured AAA is a dramatic catastrophe, and aortic emergencies constitute one of the leading causes of acute death in older adults. AAA management has been centered on surgical repair of larger aneurysms to mitigate the risks of rupture, and curative early diagnosis and effective pharmacological treatments for this condition are still lacking. Nanoscience provided a possibility of more targeted imaging and drug delivery system. Multifunctional nanoparticles (NPs) may be modified with ligands or biomembranes to target agents' delivery to the lesion site, thus reducing systemic toxicity. Furthermore, NPs can improve drug solubility, circulation time, bioavailability, and efficacy after systemic administration. The varied judiciously engineered nano-biomaterials can exist stably in the blood vessels for a long time without being taken up by cells. Here, in this review, we focused on the NP application in the imaging and treatment of AAA. We hope to make an overview of NP-assisted diagnoses and therapy in AAA and discussed the potential of NP-assisted treatment.

## Introduction

An abdominal aortic aneurysm (AAA) is a localized dilatation of the aorta related to the regional weakening of the wall structure, resulting in substantial morbidity and mortality with the aortic ruptures as complications. The disease's prevalence depends on the population studied, with reported prevalence varying between 1.4 and 12.4% (1). AAAs are generally asymptomatic and are usually diagnosed by screening or as an incidental finding. The natural history of the disease is that of slow progression and ultimate rupture (2). Ruptured AAA is a dramatic catastrophe, and aortic emergencies constitute one of the leading causes of acute death in older adults (2). Multiple trials have shown no benefit of repair of AAA at sizes <55 mm in diameter, and consequently, current guidelines advise watchful waiting for aneurysms <55 mm and preventive repair once the AAA grows >55 mm (3, 4), possibly with a slightly lower intervention threshold for repair in women. AAA management has been centered for decades on surgical repair of larger aneurysms to mitigate the risks of rupture, and remarkable progress has been achieved in both open and endovascular surgery, but curative early diagnosis and effective pharmacological treatments for this condition are still lacking (5). Screening for AAA needs more imaging techniques. Many drugs such as doxycycline and rapamycin demonstrated their efficacies in animal models. However, they showed low efficacy in clinical trials, potentially because of the lack of targeted drug delivery. A significant challenge is delivering effective dosage to local lesion sites without any adverse effects (6, 7).

Nanoscience provided a possibility of more targeted imaging and drug delivery system. Nanoparticles (NPs) are particles in the nanometer range (1–100 nm), which are more than 100 times smaller than human cells, and have biological activity and fluidity in both the internal and external blood vessel systems (8). Multifunctional NPs have recently garnered considerable attention for applications that may revolutionize imaging and drug delivery. NPs can be modified with ligands or biomembranes to target therapeutic agents' targeted delivery to the lesion site, thus reducing systemic toxicity. Furthermore, NPs can improve drug solubility, circulation time, bioavailability, and efficacy after systemic administration. Judiciously engineered NPs can enable therapy and diagnosis simultaneously (9–12). Nanoscience provides a possibility that NPs can bind specific areas like degraded elastic lamina, inflammation cells, or peptides. The varied nano-biomaterials can exist stably in the blood vessels for a long time without being taken up by cells. Several reports exist with a focus on the application of NPs for enhanced imaging of aneurysmal lesions in live animals as well as in patients. It is noteworthy, however, despite relative scarcity, that recent reports have shown exciting progress in developing NP-based approaches to treat AAA *via* targeted delivery.

Here in this review, we focused on the NP application in AAA. We summarized the NP-assisted imaging and treatment of AAA to overview the value of NP in AAA diagnosis and treatment and discuss the potential use in AAA.

## Nanoparticles in Abdominal Aortic Aneurysm Imaging

Most AAAs are asymptomatic until severe complications such as rupture occur, leading to patients' sudden death (13). On the other hand, the progression rate of AAA is highly variable among different individuals. Therefore, it is vital to develop novel imaging tools and techniques for improving the sensibility and accuracy to diagnose AAA at an early stage (10, 14). Compared with conventional contrast agents, NPs have prolonged blood circulation time, controlled biological clearance pathways, and specific molecular targeting capabilities (15, 16). Due to their modifiability and biocompatibility, NPs have been reported in several studies as an agent for AAA imaging (9, 17, 18). At present, most of NPs used as contrast agents for AAA imaging are not be modified by specific targeted ligands. However, due to the infiltration of inflammatory cells, especially phagocytes that can uptake NPs, these NPs can still be detected in AAA lesion by several imaging techniques. Compared with that, previous studies have shown that NPs that are surface-modified are prone to highly accumulate at AAA lesion and show greater imaging intensity. An overview of application of NPs in several imaging techniques may improve the understanding of NP-assisted AAA imaging (see Table 1).

**Table 1:** Nanoparticles in AAA imaging.

**Nanoparticles**	**Ligands**	**Targets**	**Imaging technique**	**Models**	**Detailed application**
AuNPs	Methoxy-polyethylene glycol-thiol chains	NA	CT	Murine models	Provide clear and durable contrast enhancement of the vasculature even at 24 h (19).
EL-AuNPs	An elastin antibody	Elastin	CT	Ang II infusion in LDLr ^−/−^ mice	*In vivo* targeting to damaged aortic elastin in mice with AAA (20).
EL-AuNPs	An antibody against fragmented elastin	Fragmented elastin	CT	Porcine pancreatic elastase-induced AAA mice	Non-destructive estimation of mechanical and geometric features of AAAs when combined with CT (21).
Monocyte/ macrophage-targeted NPs	fluorine-18 [(18)F]	Monocyte/macrophage	PET-CT	ANG II infusion in apoE^−/−^ mice	Quantitation of macrophage content in a mouse AAA model (22).
Iron oxide NPs	NA	Vascular smooth muscle cells (VSMCs)	MRI	Male Fisher rats implanted with ECM tubes	Evaluating whether magnetic cell labeling can be used to non-invasively assess the technical success of endovascular cell therapy for AAAs (23).
Human ferritin NPs (HFn)	Arg-Gly-Asp (RGD)	Vascular inflammation and angiogenesis	MRI	Ang II infusion in apoE^−/−^ mice	Enhances vascular molecular imaging by targeting both vascular inflammation and angiogenesis (24).
USPIO	NA	NA	MRI	Asymptomatic AAA patients	To distinguish those patients with more rapidly progressive AAA expansion (25).
Paramagnetic/ fluorescent micellar NPs	Collagen-binding protein (CNA-35)	Collagen	MRI	Ang II infusion combined with TGF-β neutralization mouse model	The presence of collagen was visualized by nanoparticle-enhanced MRI in AAA (26).
USPIO	NA	NA	MRI	Patients with AAA	Detection of vascular inflammation to further assess AAA rupture risk (27).
SPIO	NA	Macrophages	MRI	Ang II infusion in apoE^−/−^ mice	Detection of macrophage activities in an ANG II-induced early-stage AAA model (28).
Nanoparticles	Ligands	Targets	Imaging technique	Models	Detailed application
SPIO	NA	NA	MRI	AAA patients within two weeks after EVAR	Detection of endoleaks in patients with AAA after EVAR (29).
Iron oxide NPs	NA	Macrophages	bioluminescence (BLI) and MRI	Porcine pancreatic elastase-induced AAA mice	Exploring macrophage homing and accumulation in experimental AAA disease (30).
Core/shell Fe/iron oxide NPs	MMP peptide substrate	MMP	MRI	An Ang II-induced AAA model	Detection of MMP activity within the AAA wall, thus representing a potential non-invasive method to predict the rupture risk of AAA (31).
Magnetic NPs	NA	NA	Magnetic particle imaging (MPI)	Ang II-induced ApoE^−/−^ mice	Monitoring inflammatory progression in AAA in an experimental setting (32).

*AuNPs, gold nanoparticles; USPIO, ultrasmall superparamagnetic iron oxide; NA, not acquired; MMP, matrix metalloproteinases; ANG II, Angiotensin II; LDLr, low density-lipoprotein receptor; apoE, apolipoprotein E; EVAR, endovascular aneurysm repair*.

### Nanoparticles in Computed Tomography

CT provides a non-invasive imaging capability with higher spatial and temporal resolution but lowers sensitivity as compared with other clinical imaging methods (PET and MRI). There have been studies exploring the application of CT in the diagnosis of AAA. For example, CT signal heterogeneity measurements in small AAAs have been revealed as a useful risk stratification tool to identify aneurysms with a high risk of significant expansion (33). Gold NPs (AuNPs) have attracted much attention as an X-ray contrast agent in recent years due to their high X-ray attenuation, non-toxicity, easy synthesis, and surface functionalization (19). Compared with barium sulfate and iodine, gold shows high X-ray attenuation coefficient, especially in the energy level for clinical CT. AuNPs exhibit longer vascular residence time than iodide molecules because of their higher molecular weight, which may increase the available imaging window (34). Au et al. (35) reported that AuNPs could provide clear and durable contrast enhancement of the vasculature even at 24 h (20). Studies have shown that AuNPs could be used to effectively label several cell types, including stem cells and immune cells, without damaging their activities. This could be used as a novel method for *in vivo* tracking of therapeutic cells (21). Using an angiotensin II (ANG II)-induced AAA mouse model, Wang et al. (36) revealed *in vivo* targeting of AuNPs conjugated with elastin (EL-AuNPs) of damaged aortic elastin in AAA lesions, and a correlation between micro-CT-based signal intensities and burst pressures was found. Lane et al. also used EL-AuNPs as a diagnostic tool for evaluating unruptured AAAs. Electron dense EL-AuNPs were visualized by micro-CT, and the corresponding gold-to-tissue volume ratios quantified. The gold-to-tissue volume ratios correlated strongly with the concentration of infused porcine pancreatic elastase and therefore the degree of elastin damage (37). These findings all indicate that AuNPs could become useful contrast agents for AAA CT imaging.

### Nanoparticles in Positron Emission Tomography

PET is a non-invasive technique that is also widely used in clinical practice. Through radiolabeled tracers in target tissues, PET enables visualization of several biochemical processes effectively at the cellular and molecular levels. Since AAA pathology is associated with inflammatory cell infiltrate and enzymatic degradation of the vessel wall, there have been numerous PET imaging reports in AAA patients. PET imaging revealed increased fluorodeoxyglucose (^18^F-FDG) uptake in patients with AAA, suggesting a possible association between increased ^18^F-FDG uptake and AAA expansion and rupture (38–41). PET combined with CT rays scan (PET-CT) has also been revealed as a promising technique to identify the aneurysm wall (42–44). However, a study reported that the chronic inflammation observed on the asymptomatic AAA wall was not metabolically sufficient to cause the increased glucose metabolism detected by FDG-PET (45). In patients with sub-renal AAA, the metabolic activity level of ^18^F-FDG PET-CT was not associated with the aortic size (46, 47). Therefore, the association between ^18^F-FDG uptake and inflammation in AAA is still controversial. It is necessary to develop novel PET tracers to investigate the pathogenesis of AAA further.

Several studies reported the application of NPs as an emerging molecular imaging tool in PET imaging (22, 48). However, few studies have investigated the application of NPs in AAA PET imaging. Nahrendorf et al. developed ^18^F-FDG-labeled NPs for PET-CT imaging. These macrophage-targeted NPs labeled with fluorine-18 were used to quantify macrophages in ANG II-induced apoE^−/−^ mouse model of AAA. The *in vivo* PET signal was significantly higher in AAA compared with wild-type aorta (49). In general, our knowledge of the application of NPs in AAA PET imaging is limited, and more studies are required to explore the role of NPs in AAA PET imaging thoroughly.

### Nanoparticles in Magnetic Resonance Imaging

MRI has been widely used in clinical practice due to its non-invasive characteristic. Based on nuclear magnetic resonance (NMR), MRI can obtain three-dimensional tomographical information with high temporal–spatial resolution in whole tissue samples. The protons' alignment was generated in the body by a powerful magnetic field and then excited by a radiofrequency pulse (RF pulse), causing proton resonance. The energy is released, and the proton spins back to the equilibrium state after being removed from the RF pulse, which can be detected by MRI sensors without involving harmful radioactivity (23). T1-weighted images can be generated from T1 (longitudinal) signals, while T2-weighted images (T2WIs) can be generated from T2 (transverse) signals (50).

There have been several studies exploring the application of NPs in MRI for AAA. Deux et al. developed iron oxide NPs for labeling vascular smooth muscle cells (VSMCs), which were used to identify further magnetic cell labeling's ability in assessing the success of endovascular cell therapy for AAA. T2^*^-weighted gradient-echo images showed areas of hypointense signal within the aortic wall immediately and up to 1 month after cell therapy, indicating that MRI with magnetic cell labeling can be used to document endovascular cell delivery (51). Kitagawa et al. developed Arg-Gly-Asp (RGD)-conjugated human ferritin (HFn) iron oxide NPs, which were then proved to enhance *in vivo* MRI by targeting both vascular inflammation and angiogenesis. Histological analysis showed colocalization of RGD-HFn-Fe_3_O_4_ to both macrophages infiltrating the vessel wall and adventitial endothelial cells, with greater uptake compared with that of non-targeted HFn-Fe_3_O_4_ in AAA lesions. This provides a promising translatable MRI approach to detect high-risk atherosclerotic and aneurysmal vascular diseases (26).

Since collagen is a significant AAA component, NPs targeting collagen could be beneficial in AAA imaging (52). In a previous study, paramagnetic/fluorescent micellar NPs functionalized with a collagen-binding protein (CNA-35) were intravenously administered into C57BL/6 mice with AAA. A significant higher MR signal enhancement was observed following the injection of CAN-35 in the aneurysmal wall compared with non-specific micelles. Confocal microscopy demonstrated the precise colocalization of CNA-35 micelles with collagen-I. This study indicated that the presence of collagen could be visualized by NP-enhanced MRI (53).

Macrophage infiltration plays an essential role in AAA pathology (25, 52, 54). Due to its characteristic of accumulating macrophages, ultrasmall superparamagnetic particles of iron oxide (USPIO) have been widely used for effectively quantifying the related inflammatory processes. Several studies have been conducted to assess whether cellular inflammation areas correlated with the rate of AAA expansion. In these studies, patients with AAA were imaged using MRI before and after administration of USPIO (27, 28, 55, 56). These studies' results emphasized that accumulation of USPIO in AAA could distinguish patients with more rapidly progressive AAA expansion. According to a study comparing combined ^18^F-FDG PET-CT and USPIO-enhanced MRI, both ^18^F-FDG PET-CT and USPIO-MRI uptake identify vascular inflammation associated with AAA (29). Superparamagnetic iron oxide (SPIO) particles can be phagocytosed by macrophages after injection, which can be visualized using MRI as areas of signal loss caused by MRI susceptibility on T2WI. SPIO particles were used in a previous study as a marker to detect macrophage activities in an ANG II-induced early-stage AAA model, and the results indicated that SPIO could be used in imaging inflammation associated with AAA (31). SPIO-enhanced dynamic MRI was used as a potential alternative to contrast-enhanced CT for detecting endoleaks after endovascular aneurysm repair (EVAR) in a study. Eight of 11 endoleaks were detected by contrast-enhanced CT (8/11:73%), while 10 endoleaks were detected by SPIO-enhanced MRI (10/11:91%), indicating that SPIO-enhanced MRI could detect more endoleaks than contrast-enhanced CT (57).

Matrix metalloproteinase (MMP) activation contributes to AAA growth and rupture. Yao et al. conducted a study to evaluate the ability of a novel activatable MRI nanoprobe to target MMPs in an ANG II-induced AAA model (58). This nanoprobe is composed of a hydrophilic polyethylene glycol (PEG) coating layer immobilized on the external surface of hydrophobic core/shell Fe/iron oxide NPs (Fe/IONPs); between them, there is a grafted MMP peptide substrate. In the mice injected with MMP-Fe/IONPs, a clear signal loss on T2WI sequences in the aneurysmal region was observed. The results indicated that MRI combined with nanoprobe allows detecting MMP activity within the AAA wall, thus representing a potential non-invasive method to predict the rupture risk of AAA.

### Nanoparticles in Other Imaging Techniques

Due to the advantages mentioned above, NPs were also applied in many other techniques for AAA imaging (24). Integrin αVβ3 is expressed by monocytes and macrophages, which plays an essential role in vascular diseases such as AAA. Razavian et al. used an αV integrin-specific tracer (^99m^Tc-NC100692) to investigate integrin-targeted imaging for detecting vessel wall inflammation (30). Arg-Gly-Asp (RGD)-conjugated HFn NPs were developed to evaluate inflammation and angiogenesis in AAA disease by near-infrared fluorescence imaging. RGD-HFn showed significantly higher signal than HFn in AAAs, and histology showed that RGD-HFn colocalized with macrophages and neoangiogenesis in AAA lesions (59). Sinha et al. developed NPs as imaging agents targeting degraded elastic lamina, a consistent pathological feature in vascular diseases (9). Based on murine AAA models, Miyama et al. developed a novel combined imaging approach with bioluminescence and MRI to study macrophage homing and accumulation in AAA, which provide novel insight into the investigation on AAA biology (32). Magnetic particle imaging (MPI) is an innovative imaging method, which can detect magnetic NPs (MNPs) with high sensitivity (60). Therefore, MPI is suitable as surrogate marker for molecular targeting of vascular inflammation. The quantitative ability for mapping MNPs establishes MPI as a promising tool for monitoring inflammatory progression in AAA as assessed by a recent study (61). In general, NPs will be applied in more and more novel techniques for AAA imaging as our understanding of NPs continues to advance.

## Nanoparticles in Abdominal Aortic Aneurysm Treatment

**Figure 1:**
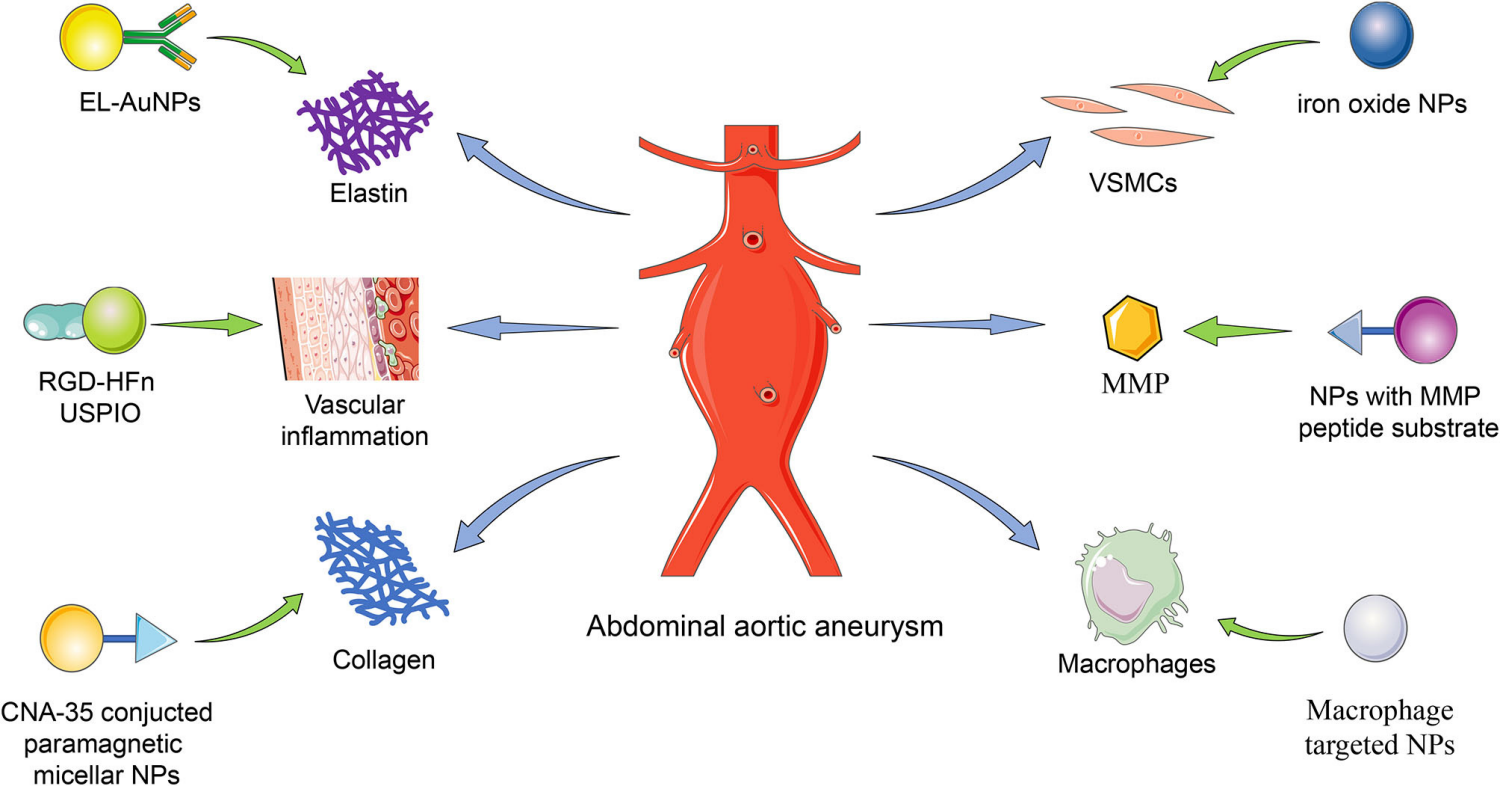
The delivery targets of NPs. The nanoparticles were delivered to the AAA lesion targeting elastin, vascular inflammation, collagen, VSMCs, MMP, and macrophage. EL, elastin; NP, nanoparticle; AuNP, gold nanoparticle; RGD, Arg-Gly-Asp; HFn, human ferritin; USPIO, ultrasmall superparamagnetic particles of iron oxide; CAN-35, collagen-binding protein; VSMC, vascular smooth muscle cell; MMP, matrix metalloproteinase.

Surgery and endovascular therapy are still the primary central management of AAA. There is no therapeutics available in the clinic for AAA treatment. Many drugs demonstrated efficacy and safety in animal models but performed severely in the clinic. A significant challenge is delivering effective dosage to local lesion sites without any adverse effects (6, 7). NP-assisted AAA treatment is based on the NP-assisted delivery approach, similar to the NP-assisted imaging technique (see Figure 1). Researchers developed NP-assisted drug delivery performance of nanocarriers for AAA treatment targeting elastase laminae and AAA lesions (see Table 2).

**Table 2:** Nanoparticles in AAA treatment.

**Nanoparticles**	**Targets**	**Component**	**Drugs loaded**	**Model**	**Administration**	**Detailed application**
EL-PEG-PLA	Elastase laminae	Elastin antibody modified PEGylated poly	BB-94	CaCl2-induced rats, Systemic calcifaction rats, aherosclerotic plaque created in ApoE^−/−^ mice	Injection once	Targeted delivery of BB-94 loaded EL-PEG-PLA NPs (8).
PLGA NPs	MMP	Coencapsulate with doxycycline and SPION, and Cationic-functionalized PLGA NPs.	Doxycycline, Statins	AngII-infusion mice, C57BL/6 mice underwent transient elastase perfusion	Implanted once; oral daily;	Inhibit MMP enzymatic function (62) and activities, and facilitate the preservation of the elastic matrix (63, 64).
HA-o-NP	VSMCs	HA loaded PLGA	HA	CaCl2-induced mice	adenoviral gene transfer	Enhanced deposition of elastin matrix and increased LOX activity (65).
Drug -loaded NPs	SMCs	TGF-β1-loaded NPs, Doxycycline-loaded NPs	TGF-β1, Doxycycline	*In vitro*		Increased elastin content and matrix assembly (66).
PEG-b-PBLG	AAA lesion	Alexa647-labeled NPs	Rapamycin	Porcine pancreatic elastase mice	IV route at 14-day and 21-day	Treating already developed AAA (67).
NP-incorporating rapamycin	AAA lesion	Poly(ethyleneglycol)-shelled nanoparticles incorporating rapamycin	Rapamycin	Porcine pancreatic elastase mice	IV route at 14-day and 21-day	Treat already developed AAA (67).
ROS-NPs	AAA lesion	PEG-phospholipid, oxidation-responsive β-cyclodextrin derivative.	Rapamycin	Porcine pancreatic elastase rats	IV route at 7-day	ROS-NPs can trigger the release of payload to control inflammation and aneurysm growth (68).

*DIR-PLA, PLA-NPs to load 1,1-dioctadecyl-3,3,3,3-tetramethylindotricarbocyanine iodide (DIR) dyes; DMAB, didodecyldimethyl ammonium bromide; DSPE-PEG, PEG-phospholipid; EL-PEG-PLA NPs, Elastin antibody-modified PEGylated poly PLA nanoparticles; LOX, lysyl oxidase; OxbCD, oxidation-responsive β-cyclodextrin derivative; PEG-b-PBLG, poly (ethylene glycol)-b-poly (γ-benzyl L-glutamate); pHEMA, biotinylated poly (2- hydroxyethyl methacrylate); RGD, Arginine-Glycine-Aspartic acid; HA, Hyaluronic acid; VSMCs, Vascular smooth muscle cells*.

### Nanoparticles Targeting the Elastic Laminae

Elastic laminae in the tunica media are formed by elastin. The elastin degradation is a pathological characteristic of aortic aneurysms. Thus, elastin is used as a target for NP (9, 62, 66–74). Sinha et al. developed an elastin antibody (EL)-modified PEGylated poly(d,l-lactide) (PLA) nanoparticles (EL-PEG-PLA NPs) binding in the elastic laminae in elastase perfusion model and CaCl_2_ model (9). They observed that EL-PEG-PLA NPs were capable of AAA targeting after systemic administration and were observed in the aortic wall's medial layer. Then they used PLA-NPs to load 1,1-dioctadecyl-3,3,3,3-tetramethylindotricarbocyanine iodide (DIR) dyes and surface maleimide groups conjugated to thiolate elastin/IgG antibodies, targeted for degraded elastin layer. DIR dyes were indelibly associated with particles to facilitate visualization and tracking of NPs (9). They found that NPs are sensitive to elastic injury and precise spatial accumulation even under high-shear hemodynamic conditions. Thus, EL-PEG-PLA NP was a predicting approach for AAA treatment when binding with certain drugs since these PLA NPs could locate diseased vascular lesson and avoid normal areas.

MMP inhibitors, which attenuate extracellular matrix (ECM) degradation, represent a promising drug class for AAA treatment. However, thus far, none of them has reached clinic success. The main limiting factors are low efficacy at low dosages but high toxicity at high dosages, quick activity loss in blood circulation, and low water solubility (7, 69). Researchers developed NP-assisted drug delivery systems that drugs can accumulate at AAA lesions depending on the limiting factors. Batimastat (BB-94) was a small-molecule inhibitor of MMPs initially developed for cancer therapy (75). The Sinha group first tested it in AAA treatment using EL-PEG-PLA NPs. Targeted delivery of BB-94-loaded EL-PEG-PLA NPs showed significantly improved efficacies in suppressing MMP activity and reducing elastin degradation, calcification, and aneurysm growth, compared with the control of free BB-94 without using EL-PEG-PLA NPs in the CaCl_2_-induced rat aortic injury model. This improvement may be explained by increased drug solubility and bioavailability yet decreased adverse effects following systemic administration using EL-PEG-PLA NPs (69).

Doxycycline is a tetracycline derivative that has also been shown to inhibit the transcription process of MMP genes. Although, doxycycline has been widely studied as a drug for AAA treatment, it showed no clear clinical benefits in AAA patients (76). Recent studies found that with local therapy *via* mini-osmotic pumps or peri-aortic foams, the doxycycline dosage sufficient to inhibit MMP activities *in vivo* could be lowered (63, 64). Sylvester et al. (67) reported the application of cationic-functionalized poly(lactic-*co*-glycolic acid) (PLGA) NPs for elastin-targeted delivery of doxycycline to AAA sites (67). Furthermore, they recently reported several new nanoplatforms for AAA treatment. The doxycycline-loaded PEG-PLGA NPs were synthesized *via* a double-emulsion solvent evaporation technique using a di-dodecyl dimethyl ammonium bromide (DMAB), a cationic surfactant, followed by PEGylation to prolong the blood circulation time and increase the water solubility (77). Other researchers used another NP platform, the PLGA NPs, to co-encapsulate doxycycline and SPION to inhibit the localized MMP and achieve magnetic field-assisted targeting for different clinical translations (78). Both designs provided desirable doxycycline release profiles (an initial burst followed by sustained release of 60 days) to inhibit the MMP activities and facilitate the preservation of the elastic matrix (77, 78). They also developed NPs for AAA tissue localized delivery of doxycycline, providing an active driving force for efficient uptake of intraluminally infused NPs to the AAA wall. Furthermore, they also rendered their polymer NPs mobile in an applied magnetic field *via* co-incorporation of superparamagnetics. The therapy can provide a non-surgical treatment option for high-risk AAA patients (78).

Statins were also loaded here in NP-assisted AAA treatment. Researchers developed a statin-loaded polymeric micelle to treat AAAs in rat models. The micelle showed medicinal efficacy by preventing aortic aneurysm expansion in a dose-dependent manner. Furthermore, the micelle-injected group showed decreased macrophage infiltration and decreased MMP-9 activity in cases of AAA (79).

### Nanoparticles Targeting Inflammation and Vascular Smooth Muscle Cell Dysfunction

NPs targeting AAA lesions can be used to treat developed AAA. Shirasu et al. reported an amphiphilic copolymer of poly(ethylene glycol)-*b*-poly(γ-benzyl-l-glutamate) (PEG-*b*-PBLG) that was synthesized and formed a micellar NP *via* self-assembly (62). Shirasu et al. used this PEG-*b*-PBLG NP system for encapsulation and targeted delivery of rapamycin. It demonstrated a preferential uptake of NPs by macrophages at AAA lesions *in vivo* and enabled effective mitigation of aortic aneurysm expansion with a rapamycin dosage as low as 0.1 mg/kg. In contrast, the free drug delivered at the same dosage failed to attenuate AAA (62). Later in 2020, Dhital et al. did further work to use a site-specific delivery system with targeted NPs to treat already developed AAA (72). They generated poly(ethylene glycol)-shelled NPs incorporating rapamycin, which has been evaluated for its potential in AAA treatment but caused many unwanted adverse effects in AAA treatment when systematically used (66, 80). Rapamycin NPs injected during the AAA formation process evinced significant suppression of AAA formation and mural inflammation, providing specific rapamycin delivery to the rat AAA and contributing to establishing a drug therapy approach targeting aortic aneurysm lesions.

Cheng et al., on the other hand, capitalized on the high oxidative stress that typically occurs in AAA lesions. They developed reactive oxygen species (ROS)-responsive and targeted nanoplatforms to deliver rapamycin. In the *in vitro* experiments, this unique design proved responsive (e.g., rapamycin release) to the H_2_O_2_ condition and inhibitory to oxidative stress and VSMC apoptosis by acting as a ROS scavenger. When administered to a CaCl_2_-induced AAA rat model, this NP conjugated with c (RGDfK) demonstrated the outstanding aneurysm-targeting ability. Furthermore, the rapamycin-loaded formulation exhibited clear therapeutic benefits, as indicated by reduced AAA size, MMP activity, macrophage infiltration, and endothelial deterioration (66).

Hyaluronic acid or hyaluronan (HA), a glycosaminoglycan (GAG) in the ECM (81), was reportedly able to stimulate elastin regeneration (82). Previous studies found that HA (HA-o) oligomers, especially the mixture of HA tetramers and hexamers, improved elastin assembly (83, 84). The negatively charged HA-o may assist elastin fiber formation through interactions with cationic tropoelastin molecules and enhanced association with versican, a large chondroitin sulfate proteoglycan commonly found in the ECM (85). However, the administration of HA-o is challenging, most notably due to their susceptibility to proteolysis in the MMP-hyperactive aneurysmal environment (65, 86) and a short half-life in the bloodstream (87). The Ramamurthi group used PLGA NPs to protect the loaded HA-o. The VSMCs treated with HA-o-NP displayed enhanced deposition of elastin matrix and increased activity of lysyl oxidase (LOX), an enzyme essential for the cross-linking of elastin fibers (88). Moreover, MMP-9 levels were reduced compared with the control of empty NP (no HA-o), whereas, MMP-2 levels were not significantly changed (67).

Transforming growth factor-beta 1 (TGF-β1), a polypeptide that regulates cell growth, differentiation, proliferation, and apoptosis (89), is also an established elastogenic factor (82, 90). TGF-β1 at higher concentrations can paradoxically induce the differentiation of SMCs to switch to an osteogenic phenotype promoting calcification (71, 91). The Ramamurthi group explored utilizing PLGA NPs to deliver TGF-β1 and doxycycline to human aortic SMCs cultured in a 3D matrix (71). The SMCs treated with TGF-β1-loaded NPs, or doxycycline-loaded NPs, showed significantly increased elastin content and matrix assembly relative to that treated with empty NP. According to these results *in vitro*, the authors accumulated that NP-assisted delivery holds the promise for TGF-β1-based therapy in AAA management (71).

## Nanoparticles in Abdominal Aortic Aneurysm Stent Graft

In addition to targeting the vascular wall, some researchers focused on the stent graft targeted delivery system. A recent publication showed a novel drug delivery system, which allowed repetitively charging a graft with therapeutic drugs and releasing them to the aortic wall *in vivo*. The system was composed of a targeted graft labeled with a small target molecule and the target-recognizing nanocarrier, which contained suitable drugs. The drug released from this drug delivery system reduced the expression of MMP-9 in mouse aortas (74). This concept would allow systemic injection of drug-loaded NPs, thus enabling a more flexible therapeutic regimen. Under this scheme, a liposome-based biotinylated NP was developed that would specifically interact with the stent graft, which is coated with biotinylated poly(2-hydroxyethyl methacrylate) (pHEMA) (74). The avidin–biotin bond has long been known to be a robust non-covalent interaction (92). In this particular application, the pHEMA stent grafts were implanted in mice to mimic post-EVAR AAA patients (74). By comparing the outcomes between the biotinylated and non-biotinylated NPs, either with or without drug encapsulation, this study demonstrated the targetability and feasibility for controlled drug release at AAA lesion site following systemic NP injection. With the ever-growing popularity of stent grafts and the persistent need for post-EVAR control of AAA progression, this particular study provides a unique concept for the future development of targeted nanoplatforms for both therapeutic and prognostic purposes.

Now, some researchers focused on the biodegradable nanofiber-loaded stent. A study *in vitro* demonstrated that the biodegradable nanofibers and the nanofiber-loaded stent graft provided sustained release of high vancomycin concentrations for up to 30 days. The *in vivo* study showed that the nanofiber-loaded stent exhibited excellent biocompatibility and released high vancomycin concentrations into the local aortic wall for 8 weeks (93).

Another study elevated the long-term outcomes of implants in small animal models. In previous research, Li et al. fabricated a poly(ε-caprolactone) (PCL) bi-layered vascular graft consisting of an internal layer with circumferentially aligned microfibers and an external layer with random nanofibers. The circumferentially oriented VSMCs were successfully regenerated after the grafts were implanted in the rat abdominal aorta for 3 months. Then they investigated the long-term (18 months) performance of the bi-layered grafts in the same model. They found that most of the grafts' luminal surface circumferentially oriented VSMCs migrated to the grafts' luminal surface to form a neointima with uniform thickness. Accordingly, ECM including collagen, elastin, and GAG displayed high density in the neointima layer while low density in the walls of the graft because of the incomplete degradation of PCL. However, the contraction and relaxation function of regenerated neoartery almost disappeared. Other factors of grafts should be considered to achieve the regenerated artery similar to the native vessels after long-term implantation, but this is still a potential orientation in AAA graft (94). What is more, combining electrospinning and 3D printing to create patient-specific nanofiber tissue-engineered vascular grafts (TEVGs) is a feasible technology as a future clinical option. Further, preclinical studies involving more complex anatomical shapes are warranted (24).

## Discussion

There have been numerous studies investigating the value of NPs in AAA imaging, treatment, and stent graft. However, although, NPs have shown high efficiency and low toxicity in these studies, there are still challenges for further clinical application. First, several animal models have been developed, such as the ANG II-induced AAA in ApoE-deficient mouse model, CaCl_2_-induced AAA model, and elastase-induced AAA model, but none of these models can accurately reproduce the pathogenic environment and disease progression of AAA patients. For instance, the complex hemodynamic changes in AAA are hard to achieve in these models. Second, the extended timing and costs, as well as potential immunogenic response, are also significant challenges to translate preclinical findings from animal models to human settings. Third, although, NPs conjugated with antibodies (e.g., EL-AuNPs) have high selectivity of AAA lesions, they are unstable in the organic solvent and are easy to denature during the conjugation process (95, 96). Compared with that, NPs conjugated with short peptides have better resistance to denaturation and have attracted more and more attention in exploring curative therapies of AAA (97).

In most of the current studies, NP-assisted imaging for AAA is based on a single imaging method. The combination of multiple imaging methods can be beneficial for improving the accuracy of AAA imaging. Combined ^18^F-FDG PET-CT and USPIO-MRI were used in a previous study for assessing tissue inflammation in AAA. ^18^F-FDG signal was focused mainly in the shoulder region of AAA, whereas, USPIO uptake was shown to be more apparent in the AAA body (29). In another study, combination of USPIO-assisted MRI and computed tomography angiogram (CTA) was used for exploring the biological and mechanical properties of AAA. The results indicated that cellular inflammation and stress may represent different but complimentary aspects of AAA disease progression (56). A combined imaging approach with bioluminescence imaging (BLI) and MRI was used to study macrophage homing and accumulation in the experimental AAA disease (32). Similar dual imaging approaches may contribute to the study of AAA biology and the evaluation of novel therapies. Therefore, future investigations should focus on the combination of several NP-assisted imaging.

The hemodynamics in the diseased abdominal aorta are considered to be a key contributor to the formation and growth of intraluminal thrombus (ILT) in AAA. In order to better understand the basic hemodynamic features in AAA imaging and seek potential treatment, many investigations have been made (98–102). Juchems et al. used computational fluid dynamics (CFD)-based blood flow simulation to analyze dynamic pressure in AAA before and after endovascular repair and provided a prognostic statement as to the possible homogenization of the pressure in abdominal stent grafts (98). Multidetector CT angiography (MDCT-A) and CFD were used to quantitatively and qualitatively assess the hemodynamic changes in AAA after stent-graft placement in another study (99). A three-dimensional flow visualization method was proposed by Chen et al. to calculate wall stress in AAA patients (100). Quantitative understanding of NPs transport and adhesion dynamic is also very challenging due to complexity of fluid dynamics, and many attempts have been made in this field (103–105). In a recent study, USPIO uptake was used to assess the wall stress in AAA. Only 16% of aneurysms exhibited colocalization of elevated stress and USPIO uptake enhancement, suggesting that cellular inflammation and stress may represent different aspects of AAA disease progression (56).

In further research, more drug-coated NP delivery systems will be developed to get an AAA-targeted local treatment. Moreover, NPs in stent graft coated with therapeutic agents or targeted components have a promising prospect in AAA treatment.

## Author Contributions

LY and KZ wrote this manuscript. ZL provided the topic of this review and performed the critical revision of the manuscript. ZL and YS contributed to the concept of this manuscript. All authors read and approved the final manuscript.

## Conflict of Interest

The authors declare that the research was conducted in the absence of any commercial or financial relationships that could be construed as a potential conflict of interest.

## Abbreviations

AAA: abdominal aortic aneurysm
CT: computed tomography
ECM: extracellular matrix
EVAR: endovascular aneurysm repair
FDG: fluorodeoxyglucose
HA: hyaluronic acid
MMP: matrix metalloproteinase
MRI: magnetic resonance imaging
NP: nanoparticle
PET: positron emission tomography
ROS: reactive oxygen species
SPIO: superparamagnetic iron oxide
USPIO: ultrasmall superparamagnetic particles of iron oxide
VSMC: vascular smooth muscle cell.
